# Chickpea Sprouts as a Potential Dietary Support in Different Prostate Disorders—A Preliminary In Vitro Study

**DOI:** 10.3390/molecules29051044

**Published:** 2024-02-28

**Authors:** Agnieszka Galanty, Ewelina Prochownik, Marta Grudzińska, Paweł Paśko

**Affiliations:** 1 Department of Pharmacognosy, Jagiellonian University Medical College, Medyczna 9, 30-688 Kraków, Poland; agnieszka.galanty@uj.edu.pl; 2 Department of Food Chemistry and Nutrition, Jagiellonian University Medical College, Medyczna 9, 30-688 Kraków, Poland; ewelina.gajdzik@uj.edu.pl (E.P.); marta.grudzinska@doctoral.uj.edu.pl (M.G.); 3 Doctoral School of Medical and Health Sciences, Jagiellonian University Medical College, 16 Łazarza Str., 31-530 Cracow, Poland

**Keywords:** chickpea sprouts, prostate cancer, benign prostatic hyperplasia, prostate disorders

## Abstract

Background: Prostate cancer (PC) and benign prostatic hyperplasia (BPH) are common health problems in the aging male population. Due to the unexplored and unconfirmed impact of food containing isoflavones, like sprouts, on the development of the management of BPH and prostate cancer, we decided to extend the knowledge in this area. Results: We have demonstrated for the first time that chickpea sprouts may play an important role in the chemoprevention of prostate disorders. However, attention should be paid to the isoflavone content in the sprouts, as in our study, chickpea sprouts with a moderate concentration of the compounds, harvested in natural light conditions (CA10L) and blue LED light (CA7B), showed the best scores in terms of their potential towards prostate disorders. Methods: Chickpea seeds were grown in LED chambers. The methanol extracts from sprouts were quantitatively defined using the HPLC system. Experiments such as the determination of PSA, 5-α-reductase, and dihydrotestosterone were performed on PNT2 and LNCaP cells. For anti-inflammatory assays (determination of NO, IL-6, and TNF-alpha release), murine RAW264.7 macrophages were used. Conclusions: The role of legume products as a diet element should be deeply evaluated for the development of future dietary recommendations for prostate cancer and BPH prevention.

## 1. Introduction

Benign prostatic hyperplasia (BPH) and prostate cancer (PC) pose significant health concerns for aging men, and their incidence is on the rise. Several environmental factors have been implicated in the onset and advancement of these conditions. Research suggests that certain dietary components may directly or indirectly impact the risk of developing BPH and PC. Additionally, factors such as obesity, smoking, and physical activity have been linked to prostate cancer [1]. Epidemiological studies increasingly point to the potential influence of specific endocrine-disrupting compounds, including pesticides, polychlorinated biphenyls, and inorganic arsenic exposure, on the development or progression of prostate disorders [2].

It is not clear if BPH increases the risk of prostate cancer development. However, a systematic review and meta-analysis published by Zhang et al. [3] suggests that there is a correlation between prostatitis, BPH, and prostate cancer.

Diet can be one of the factors that prevents or enhances the progress and development of different diseases, including prostate disorders. Rohrmann et al. [4], in a prospective study of 32,265 men in the health profession, 6092 of whom were diagnosed with BPH, found lower consumption of vegetables in men with BPH compared to their healthy counterparts. Lagiou et al. [5] conducted a study involving Greek men’s food preferences and noted an inverse association between the intake of fruits rich in β-carotene, lutein, or vitamin C and the risk of BPH, as well as an increased risk of BPH with the consumption of high-fat foods such as butter and margarine. This high-fat diet may contribute to pro-inflammatory changes in the prostate, which can lead to hypoxia and tissue remodeling [6].

Generally, the relation between diet and BPH is poorly recognized; it was noted that a decreased risk of the disease can be associated with the consumption of flaxseeds, fruits, and vegetables [6]. Nevertheless, further exploration is needed to gain a deeper understanding of the impact of micronutrients and macronutrients in conferring preventive or mitigating effects against the development of BPH.

In the case of PC, epidemiological studies and a meta-analysis show no clear relationship between dietary patterns and the risk of prostate cancer [1]. In 2018, The Japan Public Health Center-based Prospective Study showed the difference in prostate cancer risk related to three dietary patterns described as: prudent, similar to a healthy diet rich in vegetables, fruits, legumes, and fish; Western, defined by high red and processed meat consumption, potatoes, and high-fat dairy products; and traditional, which involved a characteristic Japanese diet rich in chicken, pickles, seafood, and sake [7]. This study uncovered an association between a Western diet and an elevated risk of PC in Japanese men. 

The positive influence of a healthy diet was also confirmed in Iranian men with cancer (a case-control study), but it was not observed in other studies [8].

Prins et al. [2] proposed that the observed rise in PC incidence may stem from interference with estrogen signaling, either by interacting with estrogen receptors or by affecting steroid metabolism. Notably, heightened sensitivity of this gland to estrogens has been noted during critical developmental stages (utero, neonatal, puberty). Due to such exposure, infants and children are deemed highly vulnerable to an increased risk of PC later in life [2]. Moreover, BPH stands out as the most prevalent benign neoplasm, affecting nearly 50% of men by the age of 60.

It is known that sprouts can be a significant element in the chemoprevention of hormone-dependent cancers, including prostate [9]. Chickpea sprouts are an example of functional foods rich in isoflavones [10], with a structure reminiscent of 17β-estradiol. These phytoestrogens have the capability to bind to estrogen receptors, demonstrating a stronger binding affinity for ER-β compared to ER-α. Moreover, they are more inclined to induce the transcriptional activity of ER-β, as evidenced by studies on soy isoflavones.

It can be linked to the estrogenic effect of isoflavones on prostate cells, which present a higher expression of ER-β [11]. It is known that soy isoflavone intake (genistein, daidzein) shows efficacy in preventing prostate cancer [11]. However, there is no clear evidence about the significant impact of isoflavones on BPH. In the plasma and prostatic fluid of Hong Kong men, higher concentrations of isoflavones were observed compared to those from Portugal and Britain [12]. Pejčić et al. [13] proposed that the presence of soy isoflavones, found in significant levels in the prostatic fluid of Asian men, might offer protection against various prostate disorders. However, a study involving 81 older healthy men showed no statistically significant differences in prostate-specific antigen (PSA) levels after 1 year of consuming a soy protein drink [14]. Goetzl et al. [15] investigated the effects of isoflavones (40 mg daily for 12 months) in men with BPH and lower urinary tract illnesses and noted only slight clinical improvement.

Due to such an unexplored and unconfirmed impact of food [5,16,17,18], especially legume foodstuffs and sprouts containing isoflavones, on the development of the management of BPH and prostate cancer, we decided to extend the knowledge in this area. These kinds of studies are crucial as an increasing tendency in plant-based diets rich in legumes is observed not only in vegan or vegetarian men of different ages. In our previously published paper, we described the chemopreventive in vitro effect of chickpea sprouts with different levels of isoflavones harvested in different light conditions (natural light, darkness, blue, yellow, green, and red LED lights) and sprouting times (5, 7, 10 days) on breast and prostate cancer cells of different origin. The isoflavone quantity data in selected chickpea sprout cells are presented in Table 1 to delve deeper into the effects on the prostate and enable a comparison of the results obtained in the present study. The chickpea sprouts were categorized into three groups based on their isoflavone content. The first group comprised sprouts with the highest sum of isoflavones, exceeding 720 mg/100 g dry weight (CA10L, CA7Y, CA10Y, CA7R). The second group had a moderate content of these compounds, ranging from 500 to 570 mg/100 g dry weight (CA7B, CA10B, CA10N). The third group exhibited lower isoflavone content, ranging from 110 to 320 mg/100 g dry weight (CA5N, CA10G). Notably, all sprouts harvested under blue LED light demonstrated a moderate level of active compounds, while those under yellow LED light exhibited a high amount of isoflavones. 

The data regarding the influence of different doses of isoflavones on prostate function are scarce. Therefore, the aim of the current study was to further investigate the selected chickpea sprouts regarding their anti-BPH potential in testosterone-stimulated PNT2 prostate epithelial cells in an in vitro model, as described in our previous paper [19]. We evaluated whether these sprouts’ extract, rich in isoflavones, could influence the proliferation of testosterone-stimulated cells, but also other markers of BPH, such as 5-α-reductase (5AR) activity, PSA release, and dihydrotestosterone (DHT) amount. For better insight into the problem, these markers were also evaluated in testosterone-stimulated prostate cancer LNCaP cells. Additionally, the anti-inflammatory properties of the sprouts’ extracts were determined in the RAW macrophages model.

## 2. Results and Discussion

Our previous study examined the impact of chickpea sprouts on non-neoplastic prostate epithelial PNT2 cells, as well as various cancer cells, including androgen-dependent LNCaP, androgen-insensitive DU145, and PC3 prostate cancer cells [10]. It was observed that the sprouts exhibited activity specifically against low metastatic DU145 prostate cancer cells. According to NCI classification, this chemopreventive potential can be categorized as moderate for CA5G, CA7N, CA10L, and CA10N, with IC_50_ values ranging from 102.1 to 179.8 µg/mL, or as weak for CA5N, CA5B, CA10Y, and CA10G, with IC_50_ values of 205.6–427.5 µg/mL. However, the tested extracts were, at the same time, non-toxic for normal epithelial cells PNT2. This prompted us to extend our investigations to another prostate-related health problem, namely prostate hyperplasia, and use our newly designed and previously described in vitro model based on testosterone-stimulated PNT2 cells. For this purpose, we preselect the sprouts with the highest isoflavone content and/or cytotoxic properties (Table 1). We determined not only the antiproliferative properties of the tested extracts but also other symptoms characterizing BPH, like 5AR activity, PSA release, DHT content, or inflammation marker release.

### 2.1. Proliferation

**Figure 1:**
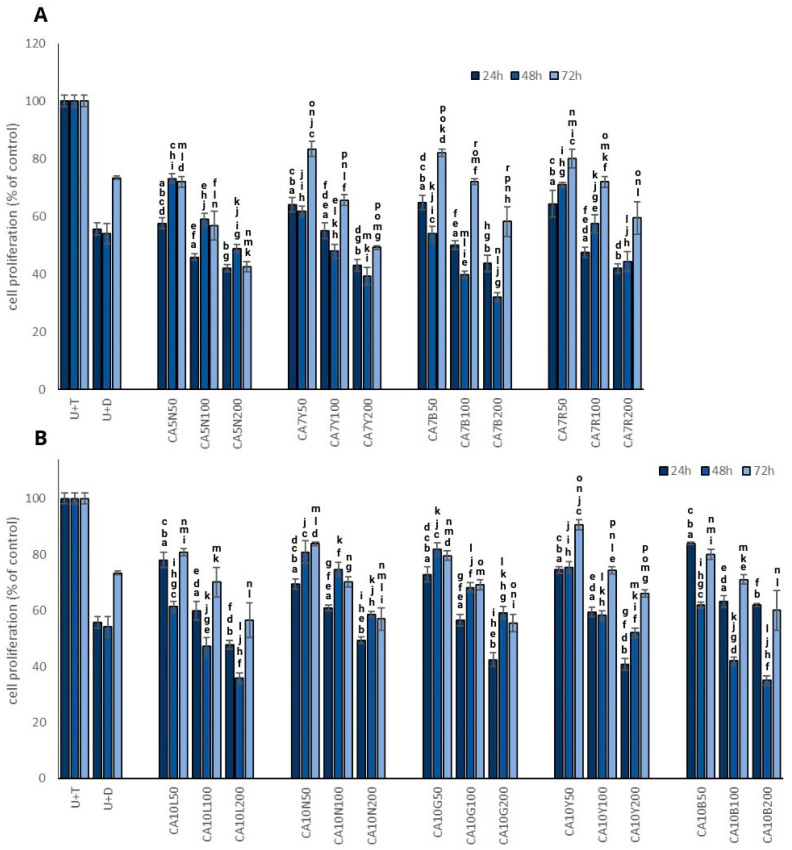
The effect of chickpea sprouts 5 and 7 DAS (**A**) and 10 DAS (**B**) in different doses (50, 100, 200 µg/mL; numbers in parentheses) on proliferation of PNT2 cells stimulated by testosterone (U + T) (*n* = 3). The data from incubation periods of 24 h, 48 h, and 72 h are individually depicted on each bar chart. They are expressed as the mean ± standard deviation (SD) of three experiments. The letters placed above each bar chart express significant differences between the results.

The most promising results in terms of the inhibition of testosterone-stimulated prostate cell proliferation were obtained for CA7B and CA10L sprouts (Figure 1A,B) in a dose-dependent manner. However, time-dependency was noted only for 24 and 48 h, with a significant increase in prostate cell proliferation after 72 h. Interestingly, a similar pattern was also noted for dutasteride, which indicates that the effect is probably transient. These two kinds of sprouts were characterized by a moderate isoflavone content (Table 1).

The mechanism of antiproliferative activity of isoflavones may be associated with apoptosis induction caused by the suppression of nuclear factor-κB through the AKT signaling pathway, which was noted in PC3 cells [20]. Lakshman et al. [21] noted that genistein may inhibit the activation of focal adhesion kinase and of the p38 mitogen-activated protein kinase (MAPK)–heat shock protein 27 (HSP27) pathway, which regulates prostate cancer cell detachment and invasion effects. It should be noted that in a mice model with orthotopic human prostate cancer cell transplant, dietary genistein suppressed cancer metastases by 96% but did not alter tumor growth in PC3-M cells. This observation, like ours, was obtained for CA10L. These sprouts were the richest source of genistein from all evaluated chickpea sprouts and significantly and dose-dependently decreased cell proliferation.

The dose-dependent manner observed in our study was confirmed by Mentor-Marcel et al. [22], who noted that dietary genistein reduced the development of prostate cancer dose-dependently in a transgenic mouse model of prostate cancer. We only found such a relationship in the case of CA10B sprouts. It is noteworthy that a soy extract containing a mixture of daidzein and genistein, along with other compounds, exhibited a more significant induction of apoptosis in PC-3 and LNCaP cells compared to daidzein or genistein [23]. Other authors suggested that soy isoflavones, particularly genistein, possess inhibitory effects on prostate cancer progression. However, they also found that a low-dose genistein diet (250 mg/kg) enhanced the growth and metastases of prostate cancer in mice compared to a control diet, while a high-dose genistein diet (1000 mg/kg) had the opposite effect [24].

Our observations clearly indicate the impact of both doses used and the time of incubation on the proliferation process. 

A meta-analysis of prospective cohort studies indicated a significant association between the high intake of legumes and the decreased risk of PC. In detail, a 3.7% decrease in the mentioned cancer risk per 20 g extra of daily legume intake was described based on a dose-response meta-analysis [25]. Diallo et al. [26] also noted an inverse relationship between PC risk and legume intake, even after excluding soy products from the study group. The findings of this study highlight a noteworthy relationship between prostate cancer and the consumption of legume products.

Matsushita et al. [1] indicate that isoflavones may also exert biphasic effects—their inadequate intake can lead to the progression of prostate cancer, which was also revealed in our study after a longer exposure. Therefore, caution should be exercised when implementing isoflavone-rich products in men’s daily diets, and more well-planned human studies should be conducted in the near future.

### 2.2. Influence of Chickpea Sprouts on 5α Reductase Activity, DHT, and PSA Release in PNT2 and LNCaP Cells Stimulated by Testosterone

Luminal cells rely on androgens, particularly dihydrotestosterone (DHT), which is a metabolite of testosterone. DHT exhibits approximately ten times higher potency due to its slower dissociation rate from the androgen receptor. Testosterone undergoes conversion to DHT by 5α reductase type 2 (5AR), an intracellular enzyme situated on the prostatic nuclear membrane in both stromal and epithelial cells. In stromal cells, DHT operates in an autocrine manner, while its diffusion into epithelial cells acts in a paracrine manner. Upon binding to nuclear androgen receptors, DHT triggers the transcription of growth factors that promote epithelial and stromal cell proliferation, ultimately leading to prostatic hyperplasia [27].

Because the 5α-reductase inhibitors markedly reduce the DHT content in the prostate and, in turn, reduce prostate volume and BPH symptoms and may be associated with the development of prostate cancer, searching for a natural element of the diet with such potential is highly needed. 

Thus, in the next step, we decided to evaluate the possible potential of selected chickpea sprouts as inhibitors of 5AR. Additionally, we used androgen-sensitive LNCaP prostate cancer cells as a reference model for the comparison of the activity of the tested extracts. 

**Figure 2:**
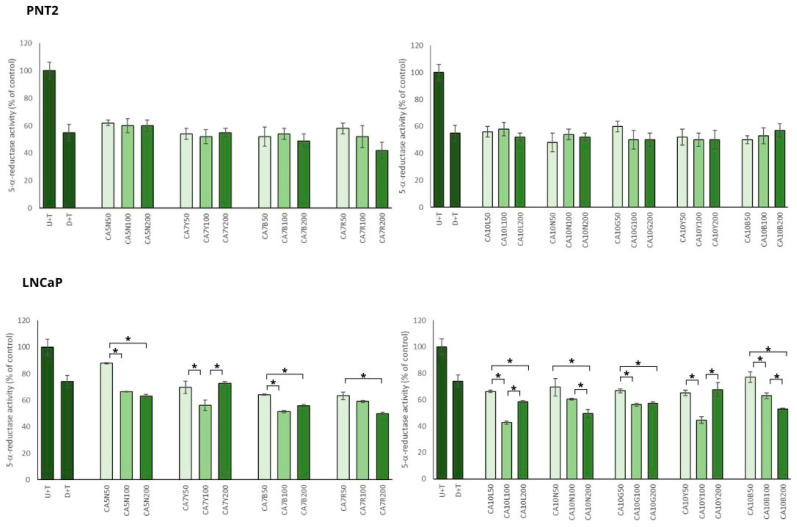
The effects of chickpea sprout extracts 5 and 7 DAS and 10 DAS on 5-α-reductase activity in PNT2 and LNCaP cells stimulated by testosterone. The cells were incubated with extract at different concentrations (50, 100, and 200 µg/mL), with reference drug dutasteride (D + T), or treated only with testosterone (U + T). The results are expressed as the mean ± SD of three experiments. Significant differences (*p* < 0.05) between tested extract doses are marked by *.

In the model of BPH with PNT2 cells, chickpea extracts showed significant inhibitory potential against 5AR, which was not dose dependent (Figure 2).

Most of the tested sprouts caused significant inhibition of 5AR in LNCaP prostate cells stimulated by testosterone (Figure 2). An interesting relationship was observed between the obtained effect on 5AR activity and isoflavone content in the sprouts. The sprouts with the highest sum of isoflavones (higher than 720 mg/100 g dw; CA10L, CA7Y, CA10Y, CA7R) caused significant inhibition of 5AR in the moderate dose used (100 µg/mL), but in the dose of 200 µg/mL, these effects were diminished and comparable to the activity obtained for 50 µg/mL. For the sprouts with a moderate content of isoflavones (from 500 up to 570 mg/100 g dw; CA7B, CA10B, CA10N), the reduction in 5AR activity was significantly dose dependent. For the sprouts with the lower content of isoflavones (from 110 up to 320 mg/100 g dw; CA5N, CA10G), a significant difference was noted between the 50 and 100 µg/mL doses, but not for the 100 and 200 µg/mL doses.

The inhibitory activity of chickpea sprouts on 5AR was more pronounced in the case of PNT2 than in LNCaP cells. Such a phenomenon could result from the overexpression of 5AR1 and 5AR2 in BPH. Furthermore, a decreased expression of 5AR2 was noted in prostate cancer cells compared with BPH [28]. This may suggest a higher effectiveness of the substances with an inhibitory effect on ARs in the prevention and treatment of low-risk prostate cancer. 

This observation was supported by DHT analysis in LNCaP cells. Significant changes in DHT levels in the prostate cancer cells treated with chickpea sprouts were noted only for the group of sprouts with a moderate concentration of isoflavones (CA7B; CA10B; CA10N) when compared to the control cells. All these sprouts caused similar changes in DHT level: for 50 µg/mL, no significant changes; for 100 µg/mL, about 20% reduction in DHT; and finally for 200 µg/mL, about 45%. Other sprout extracts revealed no significant differences in comparison to the control cells, and no influence of the doses used was noted. Contrary to our expectation, the tested extracts revealed no significant changes in DHT levels in PNT2 cells stimulated by testosterone. 

**Figure 3:**
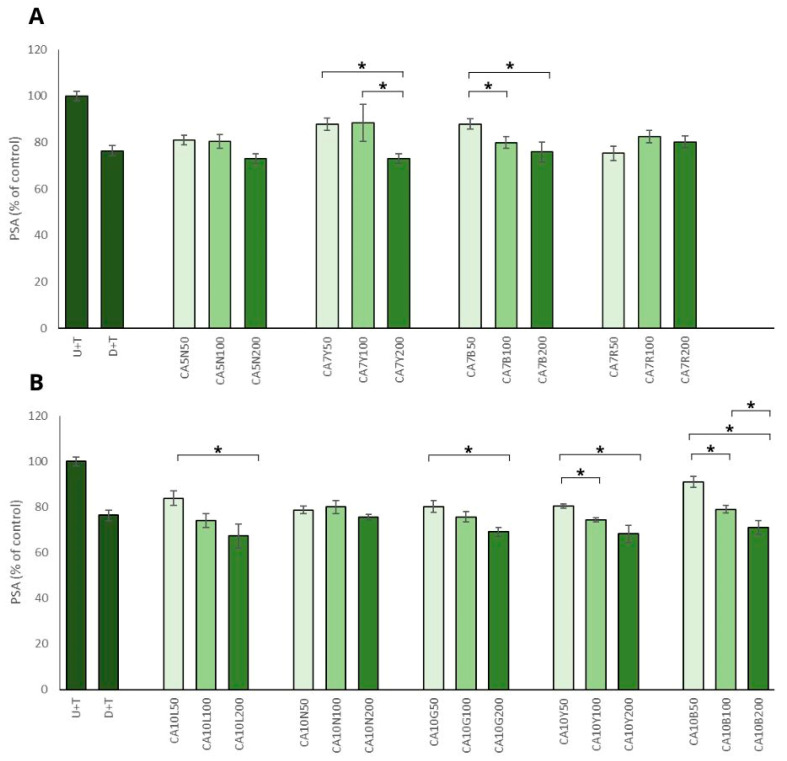
The effects of chickpea sprout extracts 5 and 7 DAS (**A**) and 10 DAS (**B**) on PSA release in LNCaP stimulated by testosterone. The cells were incubated with extract at different concentrations (50, 100, and 200 µg/mL), with reference drug dutasteride (D + T), or treated only with testosterone (U + T). The results are expressed as the mean ± SD of three experiments. Significant differences (*p* < 0.05) between tested extract doses are marked by *.

The positive impact of chickpea sprouts on prostate cancer cells was reinforced by the observed decrease in PSA level in prostate cancer cells, which was significant and mostly noted for the sprouts used in the highest doses (Figure 3A,B). The most prominent effect, namely the decrease of more than 30%, was noted for ten-day sprouts harvested in natural light conditions and also in yellow and green LED light (CA10L, CA10Y, CA10G) LNCaP cells. The remaining sprouts (CA5N, CA7Y, CA7B, CA10N, CA10B) showed a moderate (decrease by about 20%) but significant effect. This activity was not related to the isoflavone sum, but it was mostly visible for the highest doses (200 µg/mL) of chickpea sprouts. The PSA level in PNT2 cells only decreased about 10% after treatment with the examined extracts, and the differences between the samples and the doses used were not statistically significant. Our results agreed with data obtained by Wu et al. [29], who indicated that genistein and daidzein significantly inhibited AR-mediated PSA transactivation, the expression of PSA protein, and also LNCaP cell proliferation. It was proved that the two isoflavones bonded with AR as agonists, which competitively decreased the possibility of DHT binding. 

Until now, there has only been one report about the possible influence of sprouts on BPH in vitro and in vivo (rats). Song et al. [30] tested resveratrol-enriched peanut sprout extracts and indicated that these sprouts might be useful in the development of a potential BPH therapy.

At present, there are few dietary isoflavones that have demonstrated 5AR inhibitory potential, which makes them promising agents for the management of BPH, as mentioned previously, daidzein, biochanin A, equol—but it is suggested that this potential should be confirmed with in vivo models [27,31]. Bae et al. [32] searched for inhibitors of rat prostate testosterone 5α-reductase among the isoflavones, including O-methylated isoflavones. Genistein, biochanin A, equol, and 3′,4′,7-trihydroxyisoflavone revealed the highest inhibitory effects, while daidzein, formononetin, glycitein, prunetin, ipriflavone, and 4′,7-dimethoxyisoflavone were much less active. 

The importance of DHT in causing prostate hyperplasia is significant in men with BPH. The search for new 5AR inhibitors in the group of natural products that are able to reduce the DHT content in the prostate and, in turn, reduce prostate volume and BPH symptoms is highly needed. Hsu et al. [33] compared the apoptotic effects of whole soy extracts and individual isoflavones (genistein and daidzein) on benign prostate hyperplasia (BPH-1) and cancer cells (LNCaP and PC3). Soy extract triggered no changes in BPH-1 cell cycle arrest nor stimulated apoptosis, in contrast to the effect observed for both tested isoflavones, which may suggest cell-specific effects. An increase in Bax expression in PC3 cells treated with soy extract was also noted, but no significant changes in nuclear factor κB (NFκB) activation were detected. 

It is known that isoflavones can inhibit the secretion of PSA in the androgen-dependent prostate cancer cell line LNCaP [34,35]. It was also confirmed for genistein on the prostate cancer cell line VeCaP, which expresses PSA in an androgen-independent manner. Davis et al. [36] noted that genistein inhibits the growth of VeCaP cell lines and decreases PSA mRNA protein expression and secretion. These authors also observed that only high concentrations of genistein inhibited PSA expression in VeCaP cells. 

Some studies also indicated the involvement of estrogens in the etiology of BPH, associated with the age-related decrease in androgen levels in men, the increased conversion of androgen to estrogen due to higher aromatase activity responsible for this process, and subsequent stimulation of prostate cell proliferation, resulting in BPH [27,37,38]. Future research in the evaluation of isoflavone-rich dietary products on prostate function in aging men is crucial. Lewis et al. [39] published interesting results about the influence of an isoflavone-rich diet on the circulating androgen levels in 21–31-year-old Japanese men. New Zealand men who consumed a diet with low amounts of isoflavones were the control group. Genistein and equol levels in plasma, but also androstenedione, dehydroepiandrosterone sulfate (DHEAS), calculated free testosterone, and markers of 5AR, DHT, and the combined levels of androsterone sulfate and epiandrosterone sulfate (AoS/epiAoS), were significantly higher in the Japanese male group when compared to the New Zealand males. Plasma DHT and DHEAS were positively correlated with plasma equol. Similarly, plasma AoS/epiAoS and genistein levels correlated positively. Taken together, the authors suggested that the reason was the increased 5AR activity and possibly altered 17β OH steroid dehydrogenase activity in young Japanese males rather than the reduced levels of steroidogenesis. It means that the protective effect against prostate disease of dietary isoflavones may be mediated by different mechanisms according to the men’s age.

### 2.3. Influence of Chickpea Sprouts on Inflammatory Parameters

**Figure 4:**
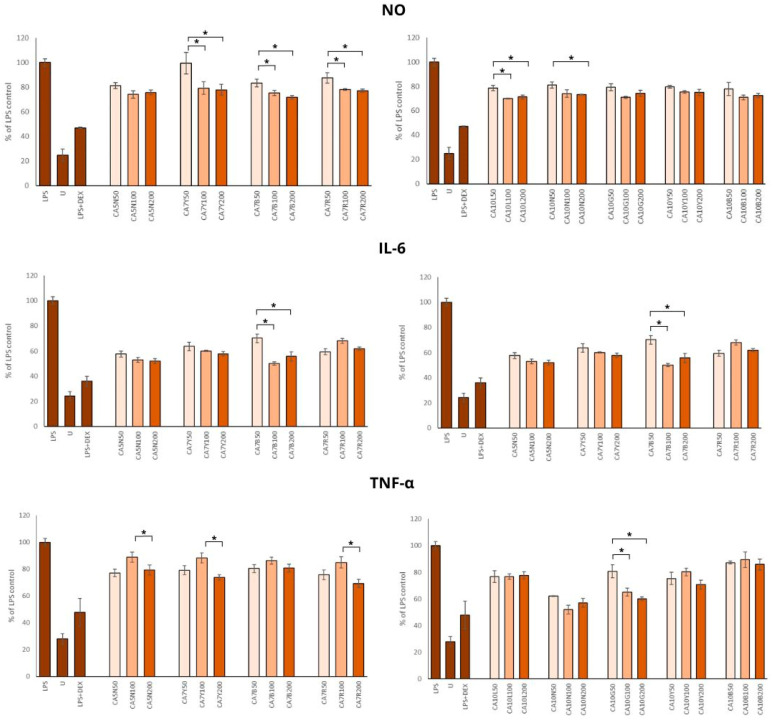
The effects of chickpea sprout extracts harvested at 5 and 7 days after seeding (DAS) and 10 DAS on the release of interleukin-6 (IL-6), tumor necrosis factor-alpha (TNF-α), and nitric oxide (NO) in lipopolysaccharide (LPS)-stimulated RAW 264.7 macrophages were investigated. RAW cells were pre-treated with sprout extract at varying concentrations (50, 100, 200 µg/mL in parentheses) for 1 h, followed by the addition of 10 ng/mL LPS to induce inflammation. Results are expressed as the mean ± standard deviation (SD) of three experiments and compared with untreated RAW cells (U) and cells treated with LPS and dexamethasone as the reference drug (DEX + LPS). Significant differences (*p* < 0.05) between tested extract doses are marked by *.

Chronic inflammation is associated with the severity and progression of benign prostatic hyperplasia and benign prostatic hyperplasia/lower urinary tract symptom outcomes. Thus, using different anti-inflammation natural agents, even in the daily diet, in patients with BPH and prostate cancer [40,41,42] should be considered. Chickpea sprouts, rich in health-promoting phytochemicals, seem to be a good candidate for such dietary support in decreasing inflammation. Our results showed a significant reduction in NO production by all sprout extracts (Figure 4). The doses of 100 and 200 µg/mL were significantly stronger inhibitors of NO synthesis than 50 µg/mL; however, no significant differences were found between the two higher doses. The highest anti-inflammatory potential was observed for CA7B, CA10B, and CA10L, which means that this activity was moderately associated with the isoflavone content in these sprouts.

Minciullo et al. [43] noted that iNOS is not detectable in normal prostates but is expressed in the prostate of all BPH patients. Milán-Noris et al. [44] evaluated the influence of chickpea isoflavones (formononetin and biochanin A; 0.5–5 mg/mL) and showed inhibitory activity on NO synthesis in RAW 264.7 macrophages stimulated with LPS. Additionally, positive correlations among the inhibitory potential of NO production and formononetin and biochanin A were noted.

The positive impact of all extracts on the IL-6 concentration was noted, but the most prominent changes were found in the group of sprouts with a moderate amount of total isoflavones (300–700 mg/100 g) (Figure 4).

This observation was only partially supported by the TNF-α level, which was reduced by all extracts in comparison to LPS control (Figure 4), but the most positive effect was noted for CA10N and CA10G, characterized by moderate and low amounts of isoflavones.

Our results on the anti-inflammatory potential of chickpea sprouts agree with Widowati et al. [45], who studied the activity of black soybean extract, daidzein, and genistein in the same macrophage model by measuring prostaglandin 2, IL-1β, and TNF-α. The concentrations used for treatments were 40 and 200 μg/mL. Of note, genistein at a concentration of 40 μg/mL showed the highest anti-inflammatory activity. Biochanin A and formononetin-rich chickpea extract administered at a dose of 10 mg/kg to rats with aluminum chloride-induced neuroinflammation inhibited the expression of such inflammatory mediators as TNF-α, NF-ĸB, and COX-2 mRNA [46].

## 3. Materials and Methods

### 3.1. Materials and Sprout Growth Conditions in LED Chambers

Chickpea (*Cicer arietinum* L.) seeds were sourced from Bavicchi Geo (Perugia, Italy). Voucher specimens were archived in the Department of Food Chemistry and Nutrition seeds collection under No. CA/PP/PL1051. The sprouting procedure was conducted according to the method described in our previous work [10]. In brief, the seeds were cultivated for 3, 5, 7, and 10 days after seeding (DAS) in: red LED 630–660 nm (R), blue LED 430–505 nm (B), green LED 550–570 nm (G), yellow LED 585–595 nm (Y), total darkness (N) (24 h/day), and natural day/night conditions (L). The samples were labeled with the chickpea Latin name acronym CA, followed by the harvesting day number and letters indicating the light conditions. 

### 3.2. Sprouts Extract Preparation and Isoflavones Analysis

The sprouts were extracted with methanol and quantitatively analyzed for isoflavone content, as described in our previous study [10]. The results are summarized in Table 1.

### 3.3. Culture Conditions

The experiments were conducted using human PNT2 prostate epithelial cell line and the androgen-sensitive prostate adenocarcinoma LNCaP cell line derived from a metastatic site (Merck, Darmstadt, Germany). For the anti-inflammatory assay, murine RAW264.7 macrophages were utilized. All cells were cultured as described previously [10].

### 3.4. Proliferation Assay

Antiproliferative activity in testosterone-stimulated PNT2 cells was measured as described previously [19,47]. 

### 3.5. Determination of PSA, 5AR, and DHT

The experiment was performed according to our previous paper [19]. The results were determined as % of control.

### 3.6. Determination of Anti-Inflammatory Potential of Chickpea Sprouts in RAW 264.7 Model

Before conducting the experiment, the impact of the tested samples on RAW 264.7 cell viability was assessed using the LDH assay, following previously established methods [48]. For the anti-inflammatory assays, RAW 264.7 cells were seeded onto 96-well plates (1.5 × 10^5^ cells/well) and pre-treated with the tested samples (50, 100, and 200 µg/mL) or dexamethasone (0.5 µg/mL), a reference drug, for 1 h. Subsequently, 10 ng/mL of LPS was added to induce inflammation, as previously described [49]. The results were expressed as a percentage of the LPS control.

### 3.7. Statistical Analysis

The data obtained were presented as mean ± standard deviation (SD). Statistical analysis was conducted using one-way analysis of variance (ANOVA), followed by a post-hoc Tukey’s test, to determine the significance of the observed differences.

## 4. Conclusions

**Figure 5:**
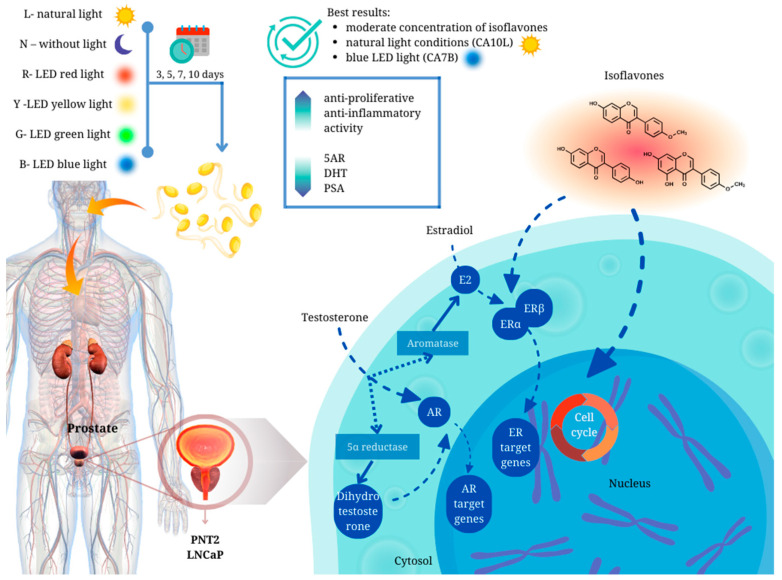
Graphical summary of the main conclusions of the article.

We have demonstrated for the first time that chickpea sprouts may play an important role in the chemoprevention of prostate disorders (Figure 5). However, attention should be paid to the isoflavone content in the sprouts, as chickpea sprouts with a moderate concentration of the compounds, harvested in natural light conditions (CA10L) and blue LED light (CA7B), showed the best scores in terms of their potential towards prostate disorders. Apart from significant antiproliferative activity, of great importance in BPH prophylaxis, these sprouts were also characterized by the significant inhibitory impact on other markers of prostate disorders, namely 5AR, DHT, and PSA, not only in testosterone-stimulated normal prostate epithelial cells but also in androgen-dependent prostate cancer cells. Importantly, CA7B and CA10L sprouts also revealed anti-inflammatory potential. Additionally, in our previous study, we showed the significant cytotoxic activity of CA10L sprouts on androgen-insensitive DU145 prostate cancer cells, making these sprouts important for further studies on other aspects of prostate disorders.

The role of legume products as an element of diet should be deeply evaluated for the development of future dietary recommendations for prostate cancer and BPH prevention.

The widespread use of a plant-based diet rich in different legume products—not only soybeans [50]—makes it important to determine the possible physiological effects in men of different ages. In addition, the presence of phytoestrogens and related estrogenic compounds in common foodstuffs affects the development and/or even therapy of hormone-dependent cancers, and this aspect should be considered in further study.

## Figures and Tables

**Table 1:** The content of individual isoflavones and their cumulative amounts in chickpea sprouts (CA) were measured in mg/100 g dw at various time points (5, 7, and 10 days after seeding—DAS) under different light conditions. The data (mean ± SD; *n* = 3) based on our prior investigation [10].

Time of Sprouting	Biochanin A	Daidzein	Formononetin	Genistein	Glycitein	Ononin	Sum of Isoflavones
CA10L	309.8 ± 29.8	3.38 ± 0.21	369.6 ± 23.5	3.02 ± 0.26	8.32 ± 0.36	97.2 ± 13.6	791.3
CA5N	100.3 ± 5.0	1.11 ± 0.06	177.9 ± 7.0	0.36 ± 0.09	2.99 ± 0.10	31.9 ± 0.7	314.6
CA10N	144.3 ± 7.9	1.57 ± 0.09	165.7 ± 14.9	1.00 ± 0.15	4.52 ± 0.16	242.5 ± 8.5	559.6
CA7R	166.9 ± 13.0	1.79 ± 0.12	182.6 ± 11.4	1.76 ± 0.04	4.46 ± 0.22	413.5 ± 16.7	771.0
CA7Y	124.8 ± 18.1	1.04 ± 0.14	136.4 ± 14.7	1.15 ± 0.11	2.61 ± 0.11	458.5 ± 27.0	724.5
CA10Y	293.9 ± 26.1	1.57 ± 0.10	227.0 ± 12.8	1.67 ± 0.15	4.11 ± 0.31	425.7 ± 6.6	953.9
CA10G	54.7 ± 5.5	0.32 ± 0.08	46.7 ± 1.4	0.15 ± 0.01	0.87 ± 0.07	11.8 ± 0.8	114.5
CA7B	182.6 ± 13.8	1.26 ± 0.14	272.3 ± 15.1	Tr	2.76 ± 0.23	54.1 ± 3.3	513.0
CA10B	257.2 ± 14.8	0.71 ± 0.16	275.1 ± 13.2	Tr	6.52 ± 0.65	23.9 ± 0.9	563.4

Abbreviations: L—natural light, N—without light, R—LED red light, Y—LED yellow light; G—LED green light, B—LED blue light; Tr—traces; 5, 7, 10—days of sprouting.

## Data Availability

The data presented in this study are available on request from the corresponding author.
